# Synergism of verbal autopsy and diagnostic pathology autopsy for improved accuracy of mortality data

**DOI:** 10.1186/1478-7954-9-25

**Published:** 2011-08-01

**Authors:** Corinne L Fligner, Jill Murray, Drucilla J Roberts

**Affiliations:** 1 Departments of Pathology and Laboratory Medicine, University of Washington School of Medicine, Box 356100, Seattle, WA, 98195, USA; 2 National Institute for Occupational Health, National Health Laboratory Service and School of Public Health, Faculty of Health Sciences, University of the Witwatersrand, PO Box 4788, Johannesburg 2000, South Africa; 3 Massachusetts General Hospital (MGH)-Pathology, 55 Fruit Street WRN 219, Boston, MA, 02114, USA

## Commentary

This series provides an important opportunity to consider how diagnostic pathology autopsy could be used in conjunction with verbal autopsy to provide more accurate cause of death and mortality data in all countries, and specifically in those countries with inadequate or nonexistent death registrations systems. For the purposes of this commentary, the term "autopsy" will denote the medical-pathology diagnostic procedure, in contrast to "verbal autopsy."

The term "autopsy" means "to see or observe for oneself," but traditional use has been reserved for the postmortem examination of a (dead) body by a physician/pathologist, in order to identify diseases and injuries and determine the cause(s) of death. This medical-diagnostic pathology procedure integrates trained observation of the external and internal body with dissection or other invasive procedures, in order to obtain tissue samples, which are evaluated by microscopy and other specialized laboratory modalities, including chemical, toxicologic, genetic, and molecular biologic analyses. Used more broadly, the term "autopsy" reflects the aggregate of procedures used for postmortem medical diagnosis or death investigation, including investigative procedures that identify information about the deceased's medical history and the circumstances and scene of his/her death.

To most pathologists and physicians, the term "verbal autopsy" seems a contradiction in terms. However, it is a clearly defined procedure, which allows classification of cause of death and cause-specific mortality by the analysis of data derived from structured interviews of family, friends, and caretakers, as well as review of any available medical records [1]. As more than two-thirds of the world's population lives and dies in countries that lack functional vital registration systems, and in which most deaths occur outside of medical facilities and are neither enumerated nor classified by cause, verbal autopsy has become the primary methodology for determining population-based cause-specific mortality [2,3]. The development of computerized algorithmic systems for determination of cause of death by analysis of verbal autopsy data is a major focus of health metrics research, and emphasis is currently focused on using the recently-completed dataset from the Population Health Metrics Research Consortium (PHMRC) project that will allow analysis of verbal autopsy data collected from more than 12,000 hospitalized patients with causes of death established by rigorous clinical criteria.

The accuracy of verbal autopsy depends in large part on the quality of the diagnostic criteria, as well as on the age of the deceased and the type of diseases that are involved. Deaths associated with nonspecific signs and symptoms are especially problematic. The recent controversy about malaria mortality in India was a newsworthy example of the difficulty of differentiating malaria-caused deaths from those due to other febrile illnesses, such as septicemia, meningitis, encephalitis, and pneumonia [4]. Other areas of poor diagnostic specificity include maternal deaths, perinatal deaths, and stillbirths. Verbal autopsy has not been validated using deaths in which diagnostic pathology autopsies have been performed to determine the cause of death.

Diagnostic pathology autopsies have long been considered the "gold standard" for cause of death determination. Although autopsy rates are generally low in many developed countries, estimated at less than 5% in US hospital deaths, studies have continued to demonstrate substantial discordance between clinically- and autopsy-determined causes of death despite technologic advances in diagnostic modalities. These discrepancies are reflected in both clinical records and death certificates. Major diagnostic error rates involving the primary cause of death have ranged from 10% to more than 30%, even in a recent study that suggested a decline in the autopsy detection of unsuspected diagnoses [5]. The extent of antemortem diagnostic workup did not predict autopsy discrepancy rates. It is still widely accepted that a properly performed autopsy and death review or investigation can provide the most accurate determination of cause of death. The contributions of autopsy to a family's understanding of a death, to the clinician's understanding of a death, to discovery of new disease processes or effects of therapy, and to medical education are well established [6-8].

Autopsy-based studies of cause of death in Africa have also confirmed a high rate of diagnostic discrepancy. In a population-based autopsy study of HIV-1 infected gold miners, discrepancies between clinical and autopsy diagnoses were high, with 51% of infections and 55% of pulmonary tuberculosis diagnosed at autopsy having been missed clinically [9]. A study in Maputo, Mozambique noted that autopsies identified more specific diagnoses than death registries, resulting in a different distribution of leading causes of death [10]. A review of the autopsy series in sub-Saharan Africa from 1992 to 2010 showed only a weak correlation between clinical diagnosis and pathologic findings in HIV-positive individuals [11]. In a unique autopsy-based study of maternal deaths, a 40% discrepancy rate between clinical and autopsy diagnoses was identified in a tertiary referral hospital in Mozambique from 2002 to 2004 [12]. Given that verbal autopsy datasets frequently do not have any medical sources of information, it is even more likely that there are substantial discrepancies between diagnostic autopsy- and verbal autopsy-determined causes of death.

A major advantage of a diagnostic autopsy is that it can identify a cause or causes of death based on pathologic tissue diagnosis. Even in deaths in which pathologic examination does not identify a definite cause of death, that information can be integrated into the death investigation process to guide the performance of additional studies and the formulation of the most likely cause of death based on all available diagnostic information. Autopsy also identifies chronic and infectious disease processes that may not be the direct cause of death, permitting assessment of prevalence for these processes. Another advantage is that the creation of tissue repositories by preservation of tissue for histologic studies may facilitate later disease discovery and characterization, allowing modification of mortality data based on medical scientific advancement.

A major impediment to the performance of diagnostic pathology autopsies is the requirement for trained pathologists and assistants and histologic laboratory infrastructure for processing tissues for microscopy and preserving tissues, not currently available in many low- and middle-resource countries, particularly in nonurban settings. However, compared with many diagnostic modalities, such as those required for radiologic imaging, autopsy is a relatively low-tech and inexpensive procedure, which utilizes the same clinical and anatomic pathology laboratory resources needed for the provision of quality medical care in these settings. Another concern is the impact of cultural and religious attitudes about death and the handling of dead bodies. As this varies by country, culture and religion, this issue will need to be addressed on a case-by-case basis. Education, discussion, respectful communication and practice, and modification and limitation of invasive procedures for community acceptance could contribute to acceptance of this postmortem diagnostic procedure.

Verbal autopsy is a valuable indirect system for establishing cause of death and cause-specific mortality, but like any clinical and historical investigation, it will misclassify a substantial number of deaths when compared to the true "gold standard" for death classification, diagnostic pathology autopsy. Too many natural disease processes have similar presentations, symptoms, and signs, and the same assumptions that result in inaccurate cause of death determination in up to 40% of physician-certified deaths will be present in verbal autopsy data. What is now needed in low- and middle-resource countries are robust studies of the concordance of verbal and diagnostic pathology autopsy to assess the contribution that diagnostic pathology autopsy can make to the quality of verbal autopsy and other mortality data. Similar studies would also be valuable in developed countries with reportedly high-quality cause of death data. Continuous evaluation of a proportion of deaths by diagnostic pathology autopsy in conjunction with verbal autopsy could promote continuous quality assessment and improvement of mortality data, and at the same time facilitate the identification of new or emerging disease processes.

## Competing interests

CLF has received grant funding from the Washington Global Health Alliance (formerly Puget Sound Partners in Global Health) for the development of a minimally-invasive autopsy for low-resource settings.

CLF, JM, and DJR received funding from the INDEPTH Network to attend the 2011 Global Congress on Verbal Autopsy: State of the Science and a post-meeting session focused on research opportunities related to diagnostic pathology autopsies and verbal autopsies in Bali, Indonesia, in February 2011.

## Authors' contributions

CLF, JM, and DJR participated in the discussion of the concepts. CLF drafted the manuscript. All authors read, revised, edited, and approved the manuscript.
